# Assessment of Awareness of Local Anaesthetic Systemic Toxicity (LAST) among Postgraduate Trainees

**DOI:** 10.1155/2022/4090444

**Published:** 2022-11-12

**Authors:** Shafiq Ali Jamaleddin Surani, Maryam Budiman, Mawaddah Azman, Raha Abdul Rahman

**Affiliations:** ^1^Department of Anaesthesiology & Intensive Care, Penang General Hospital, Jalan Residensi, 10990 George Town, Penang, Malaysia; ^2^Department of Anaesthesiology & Intensive Care, Universiti Kebangsaan Malaysia Medical Centre, Jalan Yaacob Latif, Bandar Tun Razak, 56000 Kuala Lumpur, Malaysia; ^3^Department of Otorhinolaryngology-Head, and Neck Surgery, Universiti Kebangsaan Malaysia Medical Centre, Jalan Yaacob Latif, Bandar Tun Razak, 56000 Kuala Lumpur, Malaysia

## Abstract

**Introduction:**

Although uncommon, local anaesthetic systemic toxicity (LAST) may impose fatal risk to the patients. We investigated the awareness of LAST and knowledge on local anaesthetics among our postgraduate trainees.

**Materials and Methods:**

A total of 134 postgraduate trainees from the departments of general surgery (Surgical), orthopaedic surgery (Ortho), otorhinolaryngology (ENT), obstetrics and gynaecology (OBGYN), as well as anaesthesiology and intensive care (Anaesth) were recruited. A validated questionnaire was used to assess awareness and knowledge. All participants attended a medical-education session and completed the questionnaire as preassessment and postassessment. Data were analysed, and comparisons between disciplines were conducted.

**Results:**

The trainees' awareness of LAST was overall poor at preassessment which improved almost 6-folds at postassessment. Surprisingly, only 20 (45.5%) participants from the anaesthesiology group had awareness of LAST at preassessment, and none of the participants were from surgical, orthopaedic, and obstetrics and gynaecology departments. Preassessment scores were significantly higher in the anaesth group as compared to all other groups; with a difference in the average score for Anaesth vs Surgical of 3.46 (95%, CI:2.17, 4.74), Anaesth vs Ortho of 3.64 (95%, CI:2.64, 4.64), Anaesth vs ENT of 3.43 (95%, CI:2.20, 4.67), and Anaesth vs OBGYN of 6.93 (95%, CI:5.64, 8.21). However, there was no significant difference of awareness scores between all participants at postassessment scores.

**Conclusion:**

The overall level of awareness was poor. However, the implementation of an education session significantly improved the knowledge and awareness across all disciplines.

## 1. Introduction

Appropriately administered local anaesthetics agents (LAs) allow surgical and other interventional procedures to be conducted without the sensation of pain. It even produces anaesthesia, deferring the need for general anaesthesia. The use of local anaesthetic agents is not limited to anaesthesiologists [1–6]. The advancement in techniques of neural blockade, wider recognition of the benefits, and the development of newer LAs have made their usage increasingly more common [7].

However, if improperly administered, LAs pose a serious risk of adverse events, the most devastating of which would be local anaesthetic systemic toxicity (LAST). Failure to manage this condition promptly could lead to severe morbidity and even mortality [8]. As such, any practitioner, not limited to the anaesthesiologist, who administers these agents should be familiar with the prevention, identification, and treatment of LAST. Despite the limited data, several studies have shown that knowledge of LA doses and awareness of LAST remains poor among clinicians [9–12]. The occurrence of LAST is rare, with various retrospective reviews between 1998 and 2014 showing an incidence rate between 0.87 and 1.8 per 1000 peripheral nerve blocks [13–15]. Nonetheless, this figure is considerably higher than other significant anaesthetic complications, such as malignant hyperthermia during general anaesthesia, (occurring at a rate between 1 : 5,000 and 1 : 100,000) or spinal-epidural haematoma after neuraxial anaesthesia (1 : 18,000 epidurals and 1 : 158,000 spinal blockades) [13, 16, 17].

We hypothesized that knowledge with regards to LAST is poor among our postgraduate trainees. This study was designed to assess their level of awareness and provide an education intervention aimed to improve the awareness among them.

## 2. Materials and Methods

This was a prospective, interventional study which received approval from the Research and Ethics Committee, Department of Anaesthesiology and Intensive Care, as well as the Medical Research and Ethics Committee, Universiti Kebangsaan Malaysia Medical Centre (UKMMC), (FF-2019-170). Postgraduate trainees, who were at various stages of their training in the fields of general surgery (Surgery), orthopaedic surgery (Ortho), otorhinolaryngology (ENT), obstetrics and gynaecology (OBGYN), and anaesthesiology and intensive care (Anaesth) in UKMMC, were recruited into this study.

We designed and validated a questionnaire that was used to assess the awareness of the participants of LAST for this study. The questionnaire included questions on the demographics of the intended population, need for LAs, method of administration and doses given, safety precautions taken, monitoring used during the procedure, and the participants' knowledge of LAST. Its content validation was performed by a group of surgeons and anaesthesiologists. There were six key questions, identified as “must-know” questions and must be answered correctly to be considered adequately “aware” of LAST. Correct answers were scored as 1 mark, and no marks were given for an incorrect answer. The maximum possible score was 10 marks. Following questionnaire validation, a preliminary survey involving 30 participants was conducted for reliability analysis. The calculated Cronbach's alpha was 0.864. The questionnaire of the present study is attached as Supplementary Materials (available here).

The participants attended an hour-long medical-education session (ME) on LAST that was conducted by the investigators. Prior to the presentation, participants were required to complete the questionnaire, which was regarded as a pre-test assessment. The presentation included a brief overview of the pharmacology, dose calculation and maximum recommended doses of commonly used LAs, guidelines on safe practices, the pathophysiology of LAST, identification of its signs and symptoms, and the management of LAST. At the end of the session, time was allocated for questions and answers. A poster regarding safety guidelines on the management of severe LAST based on the guideline from the Association of Anaesthetists of Great Britain and Ireland's (AAGBI's) was used as a teaching aid. Upon completion of the session, participants completed the same questionnaire and the score was recorded as post-test assessment. Participants must attend the ME presentation and both preassessment and postassessment. Participants who did not complete the ME session or presented questionnaires with incomplete or illegible answers were excluded from data analysis. Adequate awareness of LAST is defined as being able to answer correctly all the “must-know” questions. At the end of the study, the total number of participants who were able to answer the “must-know” questions correctly during the preassessment was compared against the post-test assessment. We also analysed the average total scores of all participants. Further analysis compared the scores of preassessmentand postassessment among trainees in all disciplines.

### 2.1. Statistical Analysis

The sample size was calculated using the open-source calculator “OpenEpi–Version 3” based on calculation for comparing two means. A previous study by Edwards et al. which aimed to improve LAST awareness showed that the average baseline (preassessment) scores were 3.87/14 with a standard deviation of 3.18. Average postvideo presentation scores were 6.57 with a standard deviation of 3.52 [9]. Thus, the difference in mean response was 2.7. The sample size estimated to be able to reject the null hypothesis with a probability (power) of 0.95, and a type I error probability of 0.05 was 45 subjects. A minimum of 54 subjects needed to be recruited after considering the possibility of a 20% drop-out rate.

Data obtained from this investigation were analysed using a statistical package for social sciences; SPSS version 25.0 (IBM Corp., Armonk, NY, USA) to make an inference and draw robust conclusions. A descriptive statistic of the socio-demographic characteristics was initially analysed. Frequency and percentage were reported for the distribution of categorical variables, and continuous variables were reported as the mean ± standard deviation (SD). A one-way ANOVA was used to determine the differences between the mean scores. If there was a significant p value from one-way ANOVA, a pairwise comparison by Post Hoc test was conducted. The Turkey or Games Howel Post Hoc test was chosen based on the assumption of equal variances. An independent *t*-test was used to determine the differences in the mean scores between the two groups. The paired *t*-test was used to determine the changes from prescores to postscores obtained, and the McNemar test was used when comparing a dichotomous dependent variable. Qualitative data analysis was conducted using the chi-squared or Fisher exact test when insufficient numbers were present. All comparisons with a value of less than 0.05 were considered to have a significant difference in mean scoring.

## 3. Results

**Figure 1:**
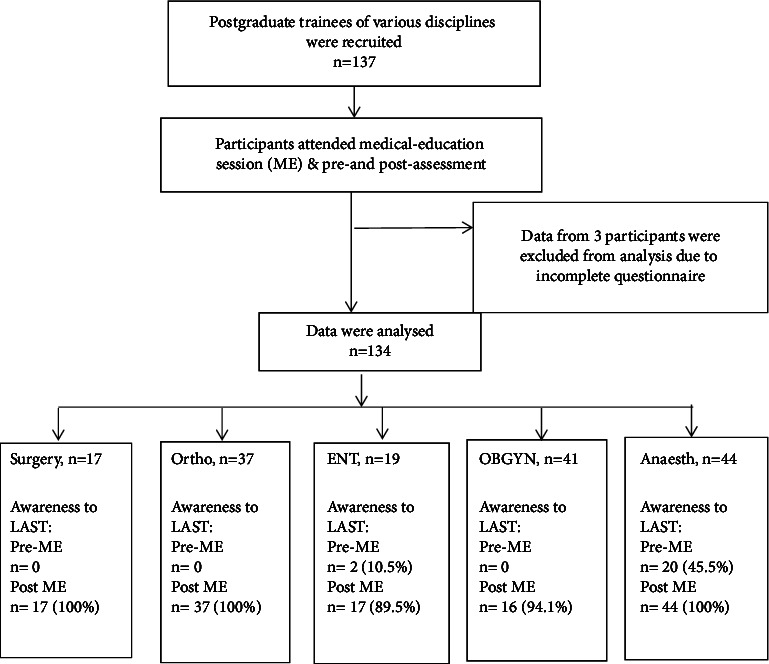
Study methodology and the primary outcome.

A total of 137 postgraduate trainees were recruited, but data from three (2.2%) participants were excluded from the analysis as the questionnaires were incompletely answered (Figure 1). There was no difference in the average years of service among the participants. However, the frequency of them using LA per week was significantly varied, reflecting the discipline that they were practicing (Table 1).

The trainees' awareness of LAST was overall poor at the preassessment (Table 2). The discipline with the highest awareness at preassessment was Anaesth (45.5%), while none of the participants from Surgical, Ortho, and OBGYN was aware of LAST. There was a marked improvement of awareness at postassessment in all groups. Hundred percent increase of awareness at postassessment in Surgical and Ortho groups, 94.1% increment in OBGYN, 79% raise in ENT, and 54.5% more participants in the Anaesth group are aware post-ME. Overall, there was almost a 6-fold increase (16.4% to 97.8%) between preassessment and postassessment (*p* < 0.001). Surprisingly, only 20 (45.5%) participants from the Anaesth group had awareness of LAST at preassessment, but the number improved to 44 (100%) following the ME session (*p* < 0.001). The awareness of participants from the Anaesth group at postassessment was comparable to the other groups (*p* = 0.551).

There was a significant improvement in average total scores of awareness, of all participants across the disciplines after they attended the ME session (Table 2). The average total improvement at post-ME session was 3.93 [95%, CI: 3.47, 4.40] marks. Further analysis using the Post Hoc pairwise comparison test showed that preassessment scores were significantly higher in the Anaesth group as compared to all other groups. The differences in the average scores for Anaesth vs Surgical was 3.46 (95%, CI:2.17, 4.74), Anaesth vs Ortho was 3.64 (95%, CI:2.64, 4.64), Anaesth vs ENT was 3.43 (95%, CI:2.20, 4.67), and Anaesth vs OBGYN was 6.93 (95%, CI:5.64, 8.21). However, there was no significant difference of awareness scores between all participants at postassessment scores, with a difference in the average score for Anaesth vs Surgical of 0.24 (95%, CI: −0.09, 0.56), Anaesth vs Ortho of 0.08 (95%, CI: −0.05, 0.21), Anaesth vs ENT of 0.37 (95%, CI: −0.05, 0.78), and Anaesth vs OBGYN of 0.18 (95%, CI: −0.12, 0.47).

**Figure 2:**
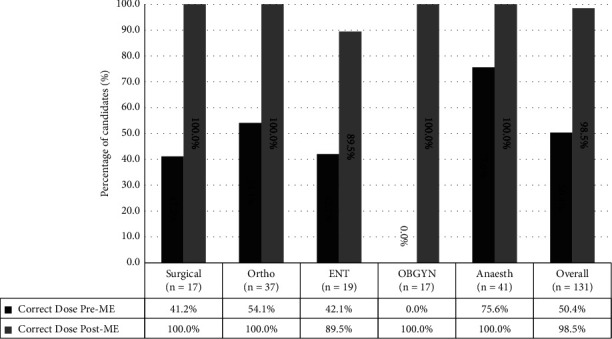
Candidates' knowledge on maximum recommended dose of lignocaine (plain and with adrenaline); comparing pre-ME results and post-ME results.

**Figure 3:**
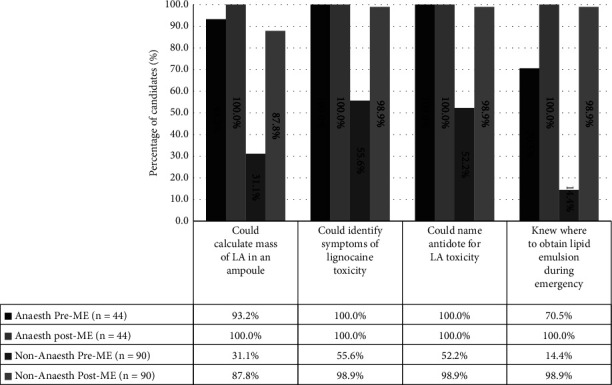
Percentage of trainees who correctly answered several questions within the questionnaire.

The most chosen local anaesthetics at preassessment and postassessment were lignocaine, 131 (97.8%). The remaining three participants who did not choose lignocaine were from the Anaesth group. We found that only 66 (50.4%) participants from the Anaesth group were able to determine the maximum recommended dose of lignocaine either as plain or when mixed with adrenaline at preassessment which later improved to 129 (98.5%) at postassessment (*p* < 0.001). On average, 31 out of 41 (75.6%) participants from the Anaesth group, and 35 out of 90 (38.9%) participants from non-Anaesth groups had knowledge of the maximum recommended dose at preassessment (Figure 2). Several other questions to assess knowledge on LA doses, symptoms of LAST, and antidote and its availability are shown in Figure 3.

## 4. Discussion

The systemic reaction to LA toxicity may cause respiratory failure, seizures, palpitations, and arrhythmias, leading to cardiac arrest and loss of consciousness which can be life threatening [8]. However, the resuscitation for LAST differs from other causes of cardiac arrest, and lack of awareness would lead to a delay or even a missed diagnosis [8]. This study found that the awareness of LAST among our postgraduate trainees was poor. Less than half of the trainees were from the anaesthesiology and intensive care discipline while almost none from the other disciplines. However, the awareness was significantly improved to near 100% after an educational session on the subject. As validated questionnaires to assess awareness have not been standardised, precise comparisons from one study to another may be difficult. Despite this, our results echoed the results of other studies involving various medical personnel, revealing poor knowledge of local anaesthetics and LAST [9–12]. An increased awareness would make the doctors more mindful with regard to their safe practices including the use of correct dose, titrating doses to effect, and adequate monitoring when administering local anaesthetics. These may reduce the incidence of LAST and mitigate rates of mortality from this life-threatening event.

It was shown that trainees from the anaesthesiology and intensive care discipline performed significantly better at preassessment when compared to the nonanaesthesiology trainees, which was similarly reported in previous studies [9, 11, 12]. However, only less than half of them had the awareness, which was expected to be 100%. The highest average usage of LA was also amongst the trainees of the anaesthesiology and intensive care discipline. This is understandable, as administration of LA is part of their daily clinical practices. As compared to other disciplines in this study, they perform central and peripheral neural blocks which use higher volumes and doses of LA and administer it to sites that pose a higher risk for LAST. Furthermore, the knowledge of LAST was emphasised in their training syllabus.

The Intralipid® (Fresenius Kabi Runcorn, UK) is the most widely studied intralipid emulsion (ILE) used as therapy in acute resuscitation of LAST. It is an emulsion of soya oil, glycerol, and egg phospholipids [8]. In our study, all trainees of the anaesthesiology and intensive care discipline compared to only half of the trainees of other disciplines were aware of ILE. This finding is similar to an earlier study by McKevith et al. [12]. However, the population in their study, besides doctors, also included operating assistants, senior nursing staff, and hospital coordinators. On the other hand, our study only assessed the awareness among doctors in the postgraduate training programme, who are clinicians that frequently administer local anaesthetics. Practically, it is very important that the ILE is kept available and easy to excess in an emergency situation. Only, a third of the trainees of anaesthesiology were aware of where ILE was kept. Our study also showed that only one third of the nonanaesthesiology postgraduate trainees were able to correctly calculate the dose of LA contained in a 2% 10 ml solution, while all trainees of anaesthesiology were able to perform the calculation. Earlier, Collins found that the anaesthetists knew the maximum recommended doses of plain lignocaine and lignocaine with adrenaline as compared to the nonanaesthetist group [11]. The familiarity of the anaesthetists to the LA could be the reason for these findings. Similarly, Sagir A. et al reported that not many nonanaesthesiologists knew the toxic dose of plain lignocaine and lignocaine with adrenaline [10].

This study showed that even a single educational session was able to significantly improve awareness about LAST among the postgraduate trainees. It has been recommended to have multiple educational sessions to improve knowledge, and the use of multiple instructional techniques is preferred compared to a single technique [18, 19]. This study did not assess the long term effect of the single educational session. However, on an average, most trainees only spend 24 to 36 months in our teaching centre.

## 5. Conclusion

Although there was better baseline awareness with the anaesthesiology postgraduate trainees as compared to nonanaesthesiology postgraduate trainees on LAST, the overall level of awareness was poor. However, the implementation of an education session significantly improved the knowledge and awareness across all disciplines. This signifies the need for implementation of content-related educational programmes to improve the knowledge of LAST among postgraduate trainees especially among the trainees of the Anaesthesiology and Critical Care programme in our centre.

## Figures and Tables

**Table 1:** Participants' demographic data and frequency of LA use.

Characteristics	Total	Surgical	Ortho	ENT	OBGYN	Anaesth	*P* value
	(*n=*134)	(*n*=17)	(*n*=37)	(*n*=19)	(*n*=17)	(*n*=44)	
Average years of service	6.22 ± 2.2	5.6 ± 2.1	6.6 ± 2.3	6.0 ± 3.1	6.9 ± 1.7	6.0 ± 1.9	0.257
Frequency of LA use							
More than 3 times a week	44 (32.8%)	1 (5.9%)	7 (18.9%)	3 (15.8%)	4 (23.5)	29 (65.9%)	<0.001
1–3 times a week	55 (41.0%)	9 (52.9%)	18 (48.6%)	11 (57.9%)	4 (23.5)	13 (29.5%)	
At least once a month	35 (26.1%)	7 (41.2%)	12 (32.4%)	5 (26.3%)	9 (52.9)	2 (4.5%)	

Values are expressed as the mean ± standard deviation or frequency (percentage).

**Table 2:** Comparing awareness and mean total scores (out of a maximum of 10) before ME and after ME.

Department	Awareness on LAST		*p* value	Mean total score (out of 10)		Average increase in total score, MD (95% CI)^*∗*^	*P* value
	Trainees who were aware pre-ME	Trainees who were aware post-ME		Pre-ME	Post-ME		
Surgical (*n* = 17)	0 (0%)	17 (100%)	<0.001	5.29 ± 1.72	9.76 ± 0.44	4.47 (3.54, 5.40)	<0.001
Ortho (*n* = 37)	0 (0%)	37 (100%)	<0.001	5.11 ± 1.79	9.92 ± 0.28	4.81 (4.20, 5.42)	<0.001
ENT (*n* = 19)	2 (10.5%)	17 (89.5%)	<0.001	5.32 ± 1.80	9.63 ± 0.60	4.31 (3.45, 5.18)	<0.001
OBGYN (*n* = 17)	0 (0%)	16 (94.1%)	<0.001	1.82 ± 1.38	9.82 ± 0.39	8.00 (7.34, 8.66)	<0.001
Anaesth (*n* = 44)	20 (45.5%)	44 (100%)	<0.001	8.75 ± 1.43	10.00 ± 0.00	1.25 (0.81, 1.69)	<0.001
Overall results (*n* = 134)	22 (16.4%)	131 (97.8%)	<0.001	5.94 ± 2.76	9.87 ± 0.36	3.93 (3.47, 4.40)	<0.001

Values are expressed as frequency (percentage) or mean ± standard deviation. ^*∗*^MD: mean difference and CI: confidence interval.

## Data Availability

The data that support the findings of this study are available on request from the corresponding author.

## Supplementary Materials

Supplementary Materials The questionnaire consists of 15 questions and is divided into four sections. The questions were on the demographics of the postgraduate trainees, need for LAs, method of administration and doses given, safety precautions taken, monitoring used during the procedure, and the participants' knowledge of LAST. There were six key questions, identified as “must-know” questions and must be answered correctly to be considered adequately “aware” of LAST. Correct answers were scored as 1 mark, and no marks were given for an incorrect answer. The maximum possible score was 10 marks.
