# Supplementary material for: Assessment of Awareness of Local Anaesthetic Systemic Toxicity (LAST) among Postgraduate Trainees
Source: Int J Clin Pract. 2022 Nov 12;2022:4090444. doi: 10.1155/2022/4090444 (PMC9675600; doi:10.1155/2022/4090444)
Supplement: Supplementary Materials — The questionnaire consists of 15 questions and is divided into four sections. The questions were on the demographics of the postgraduate trainees, need for LAs, method of administration and doses given, safety precautions taken, monitoring used during the procedure, and the participants' knowledge of LAST. There were six key questions, identified as “must-know” questions and must be answered correctly to be considered adequately “aware” of LAST. Correct answers were scored as 1 mark, and no marks were given for an incorrect answer. The maximum possible score was 10 marks. [file 4090444.f1.docx]

**APPENDIX: QUESTIONNAIRE**

This questionnaire consists of 15 questions. Kindly tick (✓) where applicable.

**Part 1 - Trainee Demographics:**

**Department:** Surgical Orthopaedics ENT O&G Anaesth Other (Please specify) _________

**Year:** 1 2 3 4

**Total years of service in your field**: ___ years

**Part 2 - Use of Local Anaesthetics:**

**1. On average, how frequently do you use Local Anaesthetics?**

More than three times a week

One to three times a week

At least once a month

At least once a year

**2. What are the routes of administration which you would normally use to administer local anaesthetic?** *(You may select more than one)*

| Central-neuraxial nerve blocks | Intravenous | Peripheral nerve blocks |
| --- | --- | --- |
| Subcutaneous infiltration | Topical | Other *(please specify)* ____________________ |

**3. Do you ask if a patient has drug allergies prior to administering your local anaesthetic?**

Always Sometimes Never

**4. Do you take informed consent prior to local anaesthetic administration?**

Always Sometimes Never

**5. Do you calculate the maximum safe anaesthetic dose before preparing the local anaesthetic?**

Always Sometimes Never

**6. Prior to injecting local anaesthetic, do you perform needle aspiration?**

Always Sometimes Never

**7. In your practise, what are the methods you use to monitor your patient when performing a surgical procedure under local anaesthesia?** *(You may select more than one)*

Blood pressure ECG GCS monitoring Pulse oximetry Respiratory rate None

**Part 3 - Knowledge of Local Anaesthetic doses:**

1. **Select TWO (2) local anaesthetics that you commonly use from the table below. For each agent you have chosen, provide the maximum recommended dose (i) without any additives, and (ii) with adrenaline.**

***(4 marks)***

| **Local Anaesthetic Agent** | **Maximum Recommended Dose** | |
| --- | --- | --- |
|  | **(i) Without any additives** | **(ii) With adrenaline** |
| **Bupivacaine**  (Marcaine®) | 1mg/kg  2mg/kg  4mg/kg  Not sure | 2mg/kg  4mg/kg  8mg/kg  Not sure |
| **Cocaine** | 1mg/kg  2mg/kg  3mg/kg  Not sure | 2mg/kg  4mg/kg  8mg/kg  Not sure |
| **Lignocaine** | 2mg/kg  3mg/kg  4mg/kg  Not sure | 3mg/kg  5mg/kg  7mg/kg  Not sure |
| **Ropivacaine**  (Naropin®) | 1mg/kg  2mg/kg  3mg/kg  Not sure | 2mg/kg  4mg/kg  6mg/kg  Not sure |

**9. You find a 10ml ampoule of local anaesthetic with a concentration of 0.5%. What is the total mass of local anaesthetic in that 10ml ampoule? *(1 mark)***

5mg 50mg 500mg Not Sure

**Part 4 - Knowledge of Local Anaesthetic Systemic Toxicity (LAST):**

**10. Have you ever heard of the term local anaesthetic systemic toxicity?** Yes No

**11. Select TWO *(2)* organ systems that are primarily affected after a toxic dose of local anaesthetic has been administered. *(2 marks)***

*(Please select only two, or ‘not sure’ if you are not sure)*

| Cardiovascular | Central nervous system | Endocrine | Gastrointestinal |
| --- | --- | --- | --- |
| Genito-urinary | Musculoskeletal | Respiratory | Not Sure |

**12. If a patient were to receive a toxic dose of lignocaine, what would be the *SEQUENCE* of signs or symptoms classically elicited with increasing plasma levels of lignocaine? *(1 mark)***

*(You may only pick ONE answer, or select ‘not sure’ if you are not sure)*

| Bradycardia | 🡪 | Chest wall rigidity | 🡪 | Respiratory depression | 🡪 | Loss of consciousness | 🡪 | Cardiac arrest |
| --- | --- | --- | --- | --- | --- | --- | --- | --- |
| Generalised pruritus | 🡪 | Vomiting | 🡪 | Angioedema | 🡪 | Hypotension | 🡪 | Shortness of breath |
| Peri-oral numbness | 🡪 | Tinnitus | 🡪 | Seizures | 🡪 | Respiratory depression | 🡪 | Cardiac arrest |
| Tinnitus | 🡪 | Chest wall rigidity | 🡪 | Respiratory depression | 🡪 | Severe headache | 🡪 | Seizures |
| Upper and lower limb numbness | 🡪 | Hypoxia | 🡪 | Hypotension | 🡪 | Respiratory depression | 🡪 | Bradycardia |
| Vomiting | 🡪 | Urinary incontinence | 🡪 | Severe hypertension | 🡪 | Loss of consciousness | 🡪 | Seizures |
| Not Sure |  |  |  |  |  |  |  |  |

**13. What is the specific antidote or therapy used to treat local anaesthetic systemic toxicity? *(1 mark)***

Intralipid® Praluent® Repatha® Not Sure

**14. Where is the antidote kept for emergency use in Universiti Kebangsaan Malaysia Medical Centre?**

***(1 mark)***

*(Please select only one)*

Emergency department Intensive Care Unit (ICU) Operating Theatre Pharmacy Not Sure

**15. Are you aware of any guidelines regarding the management of local anaesthetic systemic toxicity?**

Yes No
